# Phytoplankton Communities in the Eastern Tropical Pacific Ocean off Mexico and the Southern Gulf of California During the Strong El Niño of 2023/24

**DOI:** 10.3390/plants14091375

**Published:** 2025-05-01

**Authors:** María Adela Monreal-Gómez, Ligia Pérez-Cruz, Elizabeth Durán-Campos, David Alberto Salas-de-León, Carlos Mauricio Torres-Martínez, Erik Coria-Monter

**Affiliations:** 1 Instituto de Ciencias del Mar y Limnología, Universidad Nacional Autónoma de México, Av. Universidad 3000, Copilco, Mexico City 04510, Mexico; monreal@cmarl.unam.mx (M.A.M.-G.); dsalas@unam.mx (D.A.S.-d.-L.); 2 Instituto de Geofísica, Universidad Nacional Autónoma de México, Av. Universidad 3000, Copilco, Mexico City 04510, Mexico; perezcruz@igeofisica.unam.mx; 3 Escuela Nacional de Ciencias de la Tierra, Universidad Nacional Autónoma de México, Av. Universidad 3000, Copilco, Mexico City 04510, Mexico; elizabeth.duran@encit.unam.mx; 4 Posgrado en Ciencias del Mar y Limnología, Universidad Nacional Autónoma de México, Av. Universidad 3000, Copilco, Mexico City 04510, Mexico; mau_torresmtz@hotmail.com

**Keywords:** phytoplankton, species richness, abundance, El Niño 2023/24, Mexican Pacific, Gulf of California

## Abstract

This paper analyzes phytoplankton communities in the Eastern Tropical Pacific Ocean off Mexico (ETPOM) and the Southern Gulf of California (SGC) during the strong El Niño event of 2023/24. A multidisciplinary research cruise was conducted in the winter of 2024, during which high-resolution hydrographic data and water samples for phytoplankton cell determinations were collected at 33 sites. Additionally, satellite data were obtained to evaluate sea surface temperature and chlorophyll-*a* levels. A total of 269 phytoplankton species were identified, comprising one hundred and fifty diatoms, one hundred and twelve dinoflagellates, five silicoflagellates, one ciliate and one cyanobacteria. The dominant species included the diatom *Pseudo-nitzschia pseudodelicatissima*, the dinoflagellate *Gyrodinium fusiforme*, the silicoflagellate *Octactis octonaria*, and the ciliate *Mesodinium rubrum*. The cyanobacterium *Trichodesmium hildebrandtii* was also identified. In terms of total abundances, diatoms were the most prevalent, with 224,900 cells L^−1^, followed by dinoflagellates at 104,520 cells L^−1^, ciliates at 20,980 cells L^−1^, cyanobacteria at 1760 cells L^−1^, and silicoflagellates at 1500 cells L^−1^. Notably, interesting differences emerged in species richness and abundance when comparing both regions. These results enhance our understanding of phytoplankton dynamics associated with strong El Niño events. The ETPOM remains a region that requires further monitoring through in situ observations.

## 1. Introduction

El Niño Southern Oscillation (ENSO) is recognized as a significant ocean–atmospheric process that profoundly impacts several aspects of nature including climate patterns [1], primary and secondary production [2], fisheries [3], the economy [4], tourism [5], and the availability of freshwater [6]; it has even been linked to mass extinction events [7].

ENSO is characterized by two phases: a cold phase known as La Niña and a warm phase called El Niño. Over the past 50 years, this phenomenon has garnered considerable attention from the scientific community. Since the 1980s, there have been several notable El Niño events, including those in 1982/83, 1986/87, 1997/98, and 2015/16 [8].

The last five years have seen significant fluctuations in ENSO activity. For instance, from mid-2020 to 2022, a strong La Niña event was observed, which quickly transitioned into an intense El Niño event, comparable to that of 2015/16. In the Eastern Pacific Ocean, El Niño is associated with the movement of warm, nutrient-poor water masses, which significantly impact primary production, particularly that of phytoplankton [9].

Phytoplankton are a highly diverse and abundant group of microorganisms that play essential roles in any ecosystem. Indeed, they contribute to the release of oxygen and the sequestration of carbon through photosynthesis [10]. Additionally, as the base of the food chain, phytoplankton support numerous fisheries, some of which are of considerable commercial value [11].

Numerous studies indicate that ENSO events significantly affect the structure of phytoplankton communities. In the Pacific Ocean, El Niño events are particularly associated with major changes in the taxonomic composition of phytoplankton. During the 2015/16 El Niño, for example, the surface waters of the equatorial and northeastern Pacific Ocean experienced exceptionally warm conditions, which led to a decline of approximately 40% in surface chlorophyll-*a* levels. This decline was linked to a near-total collapse of diatom populations [12]. Additionally, a significant decrease in the abundance of haptophytes was documented during this event [13]. Furthermore, blooms of species adapted to high temperatures and low nutrient levels were observed [14]. In the Western Tropical Pacific Ocean, the 1986/87 El Niño event saw extremely low chlorophyll-*a* concentrations (<0.2 mg m^−3^), with a noticeable reduction in the abundance of phytoplankton cells, including cyanobacteria, compared to subsequent years [15].

In the Gulf of California, recent studies have shown that the structure of the phytoplankton community in the coastal region of the eastern entrance changes significantly depending on whether El Niño or La Niña conditions are present. For instance, during the La Niña event of 2022, a total of 232 phytoplankton species were observed, which included 125 diatom species, 101 dinoflagellate species, 2 silicoflagellate species, 2 euglenoid species, 1 coccolithophorid, and 1 ciliate [16]. In contrast, during the El Niño event of 2023/24, the composition of phytoplankton was notably different, with a total of 197 species identified. This included 113 diatoms, 76 dinoflagellates, 4 silicoflagellates, 2 cyanobacteria, 1 Euglenophyta, and 1 ciliate [17].

**Figure 1:**
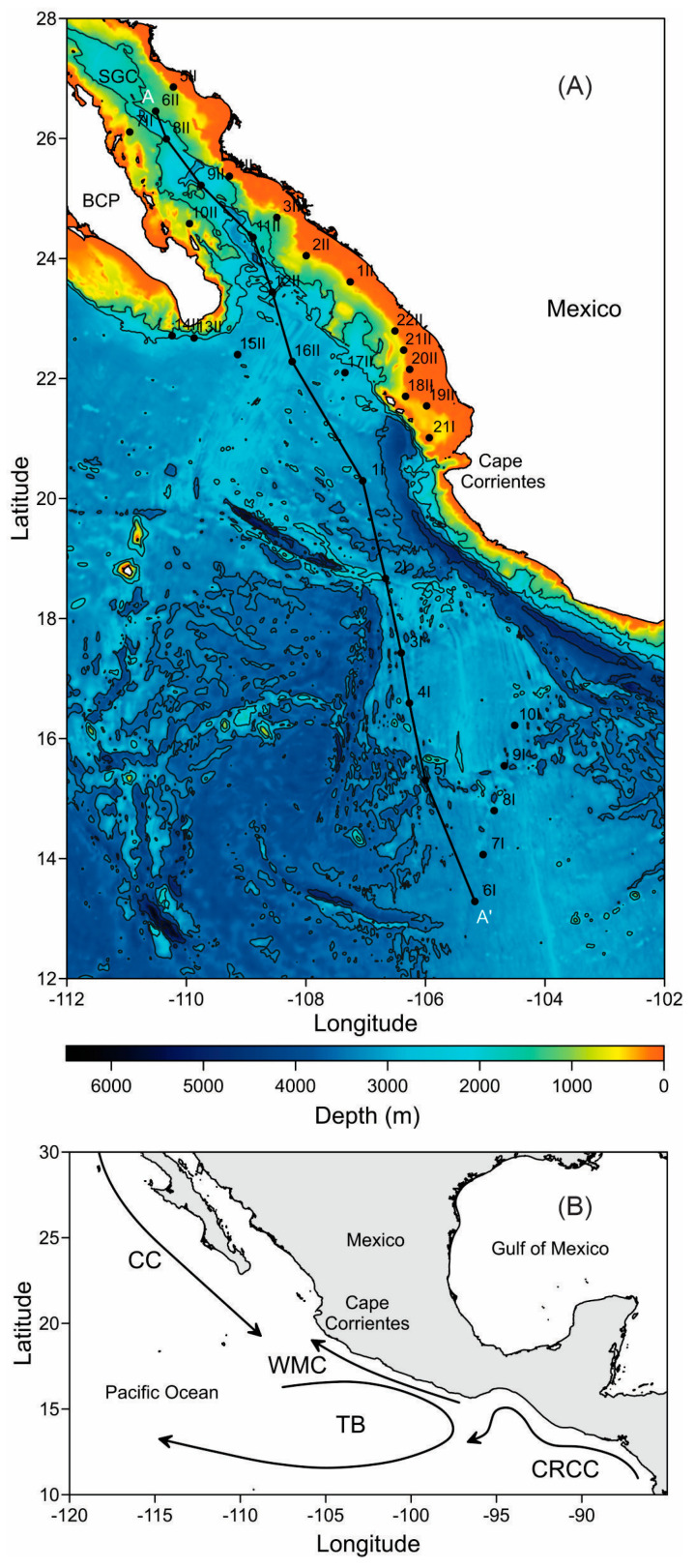
(**A**) Study area; the Eastern Tropical Pacific Ocean off Mexico (ETPOM) and the Southern Gulf of California (SGC). The • symbols represent the hydrographic stations where surface water samples were collected for phytoplankton cell determinations. A-A’ represents a transect where the vertical distribution of conservative temperature, absolute salinity and chlorophyll-*a* was analyzed. Bathymetry is displayed in meters, and (**B**) key currents occurring in the study region are the California Current (CC), the West Mexican Current (WMC), the Tehuantepec Bowl (TB), and the Costa Rica Coastal Current (CRCC) after Kesler [18].

Research on ENSO impacts on the phytoplankton populations is gaining momentum, owing to its importance in climate research. As mentioned above, the past five years have seen significant changes in the Earth’s climate system. Specifically, a strong La Niña event took place from mid-2020 to early 2023. This event was followed by a rapid transition to an El Niño episode, which recorded Oceanic Niño Index (ONI) values of 2.0 in December 2023 and 1.8 in January 2024, indicating a strong El Niño, whose consequences still need to be addressed. This study aims to assess the impact of the strong El Niño event of 2023/24 on the phytoplankton communities in the Eastern Tropical Pacific Ocean off Mexico (ETPOM) and the Southern Gulf of California (SGC) (Figure 1). To achieve our objective, we used high resolution hydrographic data and water samples collected during a multidisciplinary research cruise conducted in January and February of 2024, shortly before the event concluded. We assume this timing reflects the period of maximum influence of this event. Through this study, we aim to enhance the understanding of the phytoplankton dynamics associated with strong El Niño events based on in situ observations, which, particularly for the ETPOM, are still limited.

## 2. Results

### 2.1. The Oceanic Niño Index

By monitoring the development of the ONI, it was found that the index recorded a value of 0.5 in April, May, and June 2023. This value gradually increased, reaching 2.0 by December 2023, indicating a strong El Niño event. In the subsequent four months of 2024, the ONI values remained positive at 1.8, 1.5, 1.1, and 0.7, respectively.

### 2.2. Vertical Distribution of Conservative Temperature and Chlorophyll-a Levels Along Transect A-A′ from In Situ Data

**Figure 2:**
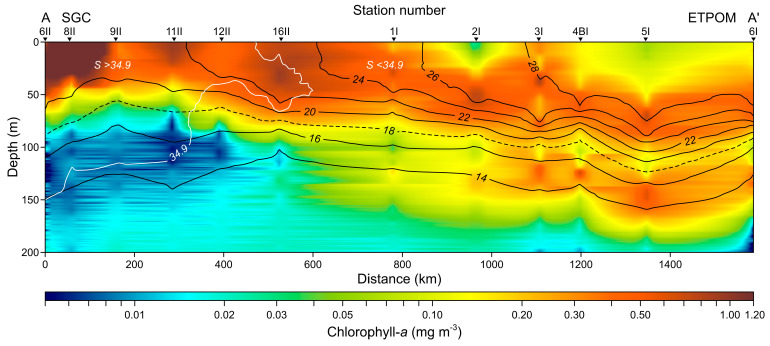
Vertical distribution of conservative temperature (°C) (black lines; isotherms) and chlorophyll-*a* (mg m^−3^) levels along the A-A′ transect in the upper 200 m layer (white line represents the isohaline of 34.9 g kg^−1^).

The vertical distribution along transect A-A′, which extends from the SGC to ETPOM (from 26.5° to 13.5° N), showed a temperature range of 13 to 29 °C in the upper 200 m layer. Sea surface temperatures decreased northward, ranging from 29° to 21 °C. There was a tendency for isotherms to uplift near station 9II and deepen near station 5I, suggesting a cyclonic and anticyclonic structure, respectively. Chlorophyll-*a* levels peaked at 1.2 mg m^−3^ in the SGC and decreased toward the ETPOM, which was associated with the deepening of the thermocline (Figure 2). One of the hydrographic characteristics of the study area during El Niño events is the presence of Tropical Surface Water (TSW) in the Gulf of California during winter. The 18 °C isotherm marks the limit of the TSW, while the 34.9 g kg^−1^ isohaline separates the Gulf California Water (GCW) from the TSW. Three water masses occupied the upper 200 m layer: TSW (T ≥ 18 °C and S < 34.9), GCW (T ≥ 12 °C and S > 34.9), and Subtropical Subsurface Water (StSsW; 9 ≤ T < 18 °C and 34.5 < S < 35). The GCW occupied the northern portion of the vertical section, delineated by the 34.9 g kg^−1^ isohaline (represented by the white line in Figure 2); from there to the southern region, the upper 100 m layer was occupied by TSW, delimited by the 18 °C isotherm, with StSsW located below them.

### 2.3. Sea Surface Temperature and Chlorophyll-a Derived from Satellite

**Figure 3:**
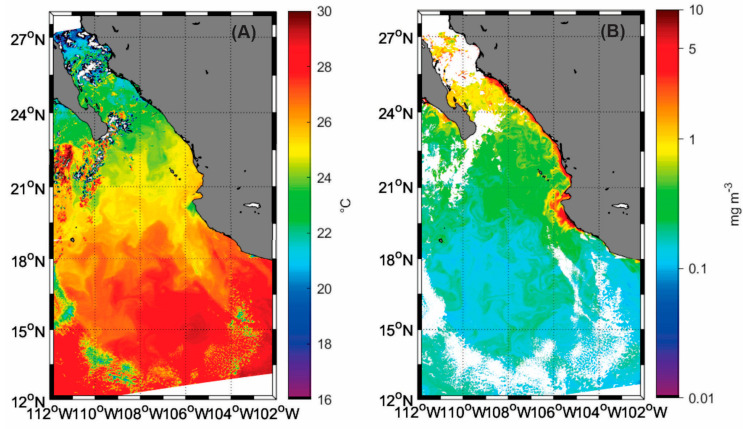
Satellite images (MODIS-AQUA) taken on 5 February 2024, during the sampling period: (**A**) sea surface temperature (°C) and (**B**) chlorophyll-*a* (mg m^−3^).

The satellite-derived images revealed interesting features. The sea surface temperature (Figure 3A) indicates a range from 18 to 30 °C, with an increase observed from the SGC toward the ETPOM. In the Gulf region, temperatures were approximately 18 °C, while in the Pacific region, they rose significantly, reaching up to 30 °C. Notably, in the southernmost region, there was a pool of warm water that covered nearly the entire southern portion.

In the case of chlorophyll-*a* (Figure 3B), the highest concentrations (up to 10 mg m^−3^) were observed along the eastern coast of the SGC. The second highest concentrations were noted in the southern region of the Gulf, while the lowest levels (~0.1 mg m^−3^) were observed in the southern part of the study area, particularly in relation to the warm water pool.

### 2.4. Phytoplankton Community Structure

The sampling strategy employed in this study enabled us to identify the phytoplankton species during the strong El Niño event of 2023/24, specifically in the ETPOM region and the SGC. A total of 269 species were identified, which included one hundred and fifty diatoms, one hundred and twelve dinoflagellates, five silicoflagellates, one ciliate, and one cyanobacterium. In terms of total abundance, diatoms were the most prevalent, with 224,900 cells L^−1^, followed by dinoflagellates at 104,520 cells L^−1^, silicoflagellates at 1500 cells L^−1^, ciliates at 20,980 cells L^−1^, and cyanobacteria at 1760 cells L^−1^. In terms of dominance, the following species were identified: the diatom *Pseudo-nitzschia pseudodelicatissima* (Hasle) Hasle 1993, the dinoflagellate *Gyrodinium fusiforme* Kofoid and Swezy 1921, the sillicoflagelate *Octactis octonaria* (Ehrenberg) Hovasse 1946, the ciliate *Mesodinium rubrum* Lohmann 1908, and the cyanobacterium *Trichodesmium hildebrandtii* Gomont 1892 (Table S1 in Supplementary Materials).

After comparing the two regions, several interesting differences were observed. Notably, there was a clear decrease in both the richness and abundance of species in ETPOM compared to SGC. For instance, the abundance of diatoms in SGC was 204,700 cells L^−1^, whereas in ETPOM it was only 20,200 cells L^−1^. Additionally, the total abundance of dinoflagellates was significantly higher in SGC than in ETPOM. There were also differences in the dominance of diatom and silicoflagellate species between the two regions (Table S2 in Supplementary Materials).

### 2.5. Phytoplankton Horizontal Distribution

After identifying the dominant phytoplankton species (Tables S1 and S2, see Supplementary Materials), we analyzed their horizontal distribution.

**Figure 4:**
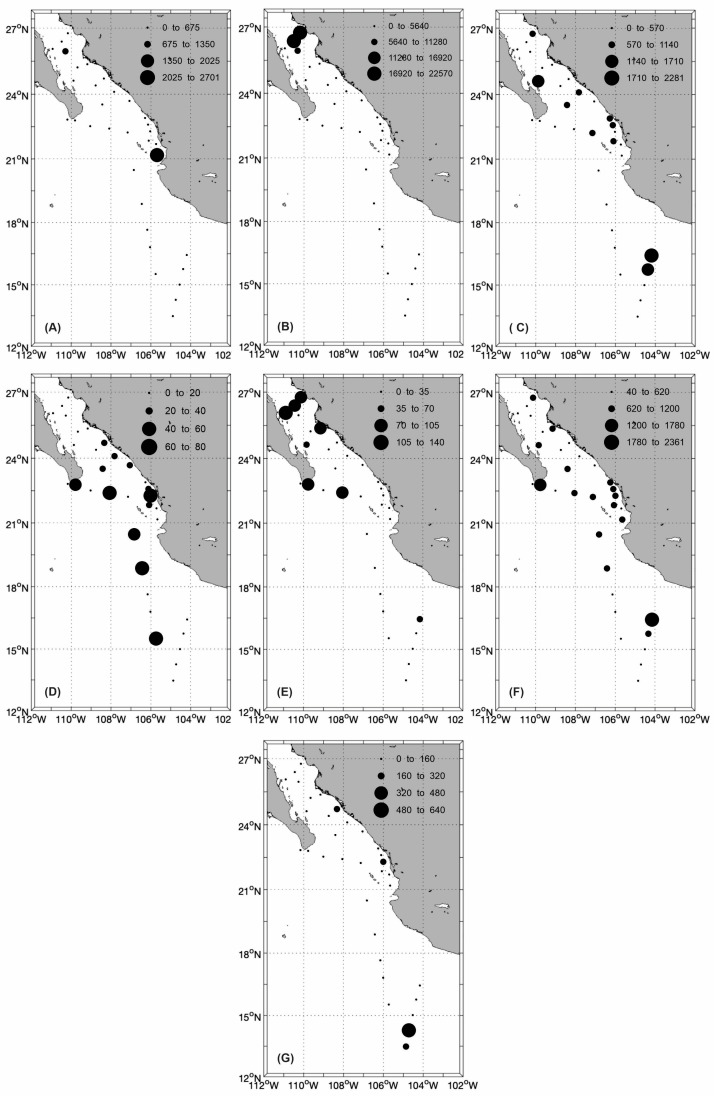
Horizontal distribution of the abundance (cells L^−1^) of the dominant phytoplankton species in the Eastern Tropical Pacific Ocean off Mexico (ETPOM) and Southern Gulf of California (SGC) during the strong El Niño of 2023/24: (**A**) *Guinardia striata*, (**B**) *Pseudo-nitzschia pseudodelicatissima*, (**C**) *Gyrodinium fusiforme*, (**D**) *Dictyocha fibula*, (**E**) *Octactis octonaria*, (**F**) *Mesodinium rubrum*, and (**G**) *Trichodesmium hildebrandtii*.

The results revealed notable differences in species abundance between the ETPOM and the SGC. For instance, the diatom *Guinardia striata* (Stolterfoth) Hasle 1996 (Figure 4A) exhibited its highest abundance in the ETPOM, particularly near the west coast of Mexico. In contrast, the diatom *Pseudo-nitzschia pseudodelicatissima* had its peak abundance within the Gulf, specifically in the northern part of the study area (Figure 4B). The dinoflagellate *Gyrodinium fusiforme* displayed two areas of high abundance: one in the ETPOM and another in the western section of the Gulf (Figure 4C). The silicoflagellate *Dictyocha fibula* Ehrenberg 1839 (Figure 4D) also showed maximum abundance in the ETPOM, aligning with the warm and oligotrophic water pool illustrated in Figure 3. Conversely, the silicoflagellate *Octactis octonaria* (Figure 4E) peaked in abundance both within the Gulf and at its connection to the ETPOM. The ciliate *Mesodinium rubrum* (Figure 4F) was most abundant in the ETPOM region, correlating with the warm oligotrophic water pool. A similar trend was observed for the cyanobacterium *Trichodesmium hildebrandtii*, which also showed high abundance in the ETPOM (Figure 4G).

**Figure 5:**
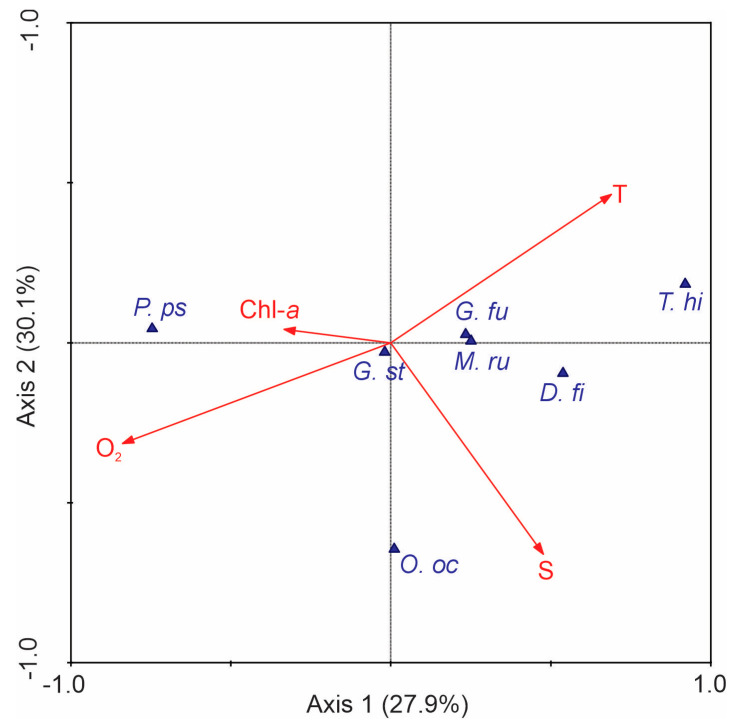
Canonical Correspondence Analysis diagram. Triangles in blue indicate the dominant phytoplankton species as illustrated in Figure 4. Vectors in red indicate environmental variables. Abbreviations are *G. st* = *Guinardia striata*, *P. ps* = *Pseudo-nitzschia pseudodelicatissima*, *G. fu* = *Gyrodinium fusiforme*, *D. fi* = *Dictyocha fibula*, *O. oc* = *Octactis octonaria*, *M. ru* = *Mesodinium rubrum*, *T. hi* = *Trichodesmium hildebrandtii,* O_2_ = dissolved oxygen, Chl-*a* = Chlorophyll-*a*, T = conservative temperature, and S = absolute salinity. The diagram showed that 58% of the accumulated variance was explained by the first two axes.

The results from the CCA revealed a strong relationship between specific variables and some species (Figure 5). For example, *G. fusiforme*, *M. rubrum*, *T. hildebrandtii*, and *D. fibula* showed a direct association with conservative temperature, while *P. pseudodelicatissima* and *G. striata* showed links to chlorophyll-*a* levels. *O. octonaria* displayed a close relationship with absolute salinity. Notably, the species associated with conservative temperatures were more abundant in the ETPOM (Figure 4), especially near the warm pool (Figure 3A). Therefore, conservative temperature is the key factor influencing the distribution of these phytoplankton species.

## 3. Discussion

The results presented here enabled us to identify the species occurring during the strong El Niño 2023/24 event in the waters of the ETPOM and the SGC, as well as to determine the horizontal distribution patterns of the dominant species. In addition, our study allowed us to identify the behavior of hydrographic variables (basically conservative temperature) and chlorophyll-*a* levels during this event.

The vertical distribution of conservative temperature and chlorophyll-*a* levels revealed notable and contrasting conditions between the SGC and the ETPOM. In the SGC, chlorophyll-*a* concentrations were quite high, exceeding 1.2 mg m^−3^ within the first 50 m of depth. However, these concentrations decreased significantly toward the ETPOM region, dropping to levels below 0.2 mg m^−3^. Interestingly, the isotherms demonstrated a pronounced subsidence in the ETPOM, aligning with the traditional understanding that the thermocline typically sinks during an El Niño event [19]. Moreover, it is worth noting that the low surface chlorophyll-*a* levels observed at stations 3I, 4BI, 5I, and 6I in the ETPOM region agree with the warm water pool illustrated in the satellite image (Figure 3A).

Regarding phytoplankton communities, our results showed a dominance of diatoms over other phytoplankton groups in both regions, which contrasts with previous studies that reported a near-total collapse of diatom populations during strong El Niño events [12]; nonetheless, in the coastal region of the eastern entrance of the Gulf, a dominance of diatoms was recently reported during El Niño 2023/24 [17], agreeing with our results.

The dominant species in our research was *Pseudo-nitzschia pseudodelicatissima* (reaching 57,000 cells L^−1^), a cosmopolitan diatom known to form harmful algal blooms and produce domoic acid [20]. Similar patterns of high abundances have been observed in different environments during El Niño conditions. Indeed, off the coast of Colombia in the Pacific Ocean, elevated abundances of this species were recorded between January and February during the 2006/07 El Niño event, averaging 21,964 cells L^−1^ in the surface waters. This increase was linked to rising surface temperatures, which resulted in a deeper thermocline [21], as was our case. Likewise, during the 1997/98 El Niño, a remarkably high abundance of this species (over 100,000 cells L^−1^) was noted in the Pacific coastal waters off Washington [22]. This was associated with an increase in surface temperatures and a weakening of the northerly winds, which usually stimulate upwellings in the region [22]. Furthermore, studies have shown that elevated temperature levels in California’s coastal waters can lead to harmful algal blooms caused by this species [23]. Therefore, the high temperatures observed during our sampling period, as depicted in the satellite image (Figure 3A), may explain the dominance of *Pseudo-nitzschia pseudodelicatissima* in our study.

*Gyrodinium fusiforme* was the dominant dinoflagellate in this study, agreeing with a recent study that addresses the phytoplankton community structure in the Mazatlán coastal region, at the eastern entrance of the Gulf [17]. This species has been reported to be highly abundant in the Pacific Ocean off Mexico and in the interior of the Gulf of California, particularly during El Niño events. Indeed, in Acapulco Bay, abundances exceeding 8500 cells L^−1^ have been documented during years affected by El Niño [24]. In Concepción Bay (Gulf of California), high abundances of this species were observed in January and February 1992, which was also an El Niño year [25]. The trend of increased abundance of this species during El Niño years has been noted in other regions as well, including the South Pacific Ocean off northern Chile [26], on the eastern Pacific coast of Ecuador [27], and in Hong Kong [28]. *G. fusiforme* is a heterotrophic dinoflagellate, which means it does not rely on nutrient concentrations for its growth [29]. In our study, we observed satellite-derived chlorophyll-*a* data, indicating very low values, especially in the oceanic region (Figure 3B). This suggests the presence of water masses with low nutrient levels [30]. Consequently, in these warm and oligotrophic waters characterized by low concentrations of chlorophyll-*a*, heterotrophic communities thrive [31]. This condition explains the dominance of this dinoflagellate in our findings.

Our study also revealed an interesting distribution pattern for the species *Dictyocha fibula*, with its highest abundances recorded in the ETPOM, particularly near the warm water pool (Figure 4D). *D. fibula* is a planktonic silicoflagellate that has been documented in the Pacific Ocean off the coast of Mexico and in the Gulf of California. This species is known to be sensitive to temperature changes [32]. Research indicates that the typical distribution of silicoflagellates in the Gulf of California is influenced by warming events. For instance, during the El Niño event of 1982/83, the influx of unusually warm water masses from the Pacific Ocean into the Gulf caused a northward displacement of this species. Furthermore, high abundances of *D. fibula* were observed in water masses that experienced temperature increases greater than 2 °C [33]. In this context, the high abundances recorded for *Octatis octonaria* (Figure 4E) can be explained by the mechanisms described in Pérez-Cruz and Molina-Cruz [33]: the advection of unusually warm water masses displaces the species northward of the Gulf, thereby influencing their horizontal distribution ranges. In fact, *O. octonaria* was reported at the SGC in El Niño 2023/24 [17].

During El Niño events, the abundance of dinoflagellates generally decreases in comparison to other groups of phytoplankton [34], which was confirmed in our study. Notably, in our study, despite its low abundance (<280 cells L^−1^), the genus *Tripos* (formerly known as *Ceratium*) contributed significantly, with a total of 11 species identified (see Table S1 in Supplementary Materials). This genus is recognized as an indicator of warm and oligotrophic water masses typically associated with El Niño events [34]. Similar conditions have been reported in various tropical and subtropical environments, including the Eastern North Pacific [35], the Pacific Ocean off Colombia [36], and the Gulf of California [37]; our findings align with those authors.

As final remarks, the impact of El Niño events, particularly in the SGC, remains a topic of ongoing debate. Some studies have reported significant negative effects on the planktonic ecosystem [33,38]. Conversely, other research suggests that several hydrodynamic processes, such as internal waves, fronts, eddies, and upwelling, may act as protective mechanisms, shielding the Gulf from the adverse impacts associated with El Niño [39,40,41]. This protective effect might explain our findings. Although we observed a significant decrease in both species’ richness and abundance in the ETPOM associated with a pool of warm water, the SGC exhibited high levels of richness and abundance, particularly of diatoms (Table S2 in Supplementary Materials). This observation aligns with results from previous studies. It appears that the several processes occurring in the Gulf could mask the negative effects of strong El Niño events; however, further observations are needed to validate this hypothesis.

## 4. Materials and Methods

### 4.1. Study Area

This study focuses on two dynamic regions that, while interconnected, are quite contrasting: the ETPOM and the SGC.

The Pacific Ocean is the largest body of water on Earth, covering more than 160 million square kilometers. The section that corresponds to Mexico, the ETPOM, spans over 2 million square kilometers (Figure 1). This region features a complex system characterized by variable bathymetry, with depths exceeding 5000 m [42]. Hydrodynamically, the ETPOM is defined by the confluence of interconnected current systems that exchange surface and subsurface water masses, each with distinct physical, chemical, and biological characteristics. Key currents include the California Current (CC), the West Mexican Current (WMC), the Tehuantepec Bowl (TB), and the Costa Rica Coastal Current (CRCC) [18]. Additionally, the ETPOM hosts several hydrodynamic processes at different scales that contribute to the region’s biological productivity. Notable examples include the upwelling of the Gulf of Tehuantepec [43] and ENSO events [44]. From a biological perspective, the ETPOM is recognized as a highly productive system that serves as a habitat for numerous species. Some of these species are threatened or endangered, while others are of significant commercial value, supporting important fisheries for shrimp, tuna, and sharks [45].

The Gulf of California is an elongated interior sea situated between the Baja California peninsula and the mainland of Mexico (Figure 1). It has a highly variable topography, with depths exceeding 3000 m in the southern region and less than 200 m in the north [41]. This area is well known for its remarkable biological diversity and serves as a habitat for many iconic species, some of which are critically endangered [46]. The Gulf is characterized by high hydrodynamism, with several processes occurring on both the microscale (e.g., internal waves and hydraulic jumps) and mesoscale (e.g., eddies and upwellings) [47]. The SGC is particularly significant scientifically, as it is directly connected to the Pacific Ocean. This connection means that many processes occurring in the southern region have important implications for the entire Gulf. One key process is the ENSO, which has notable effects on the region. This phenomenon appears within the Gulf every two years during La Niña events and has a recurrence interval of three to four years during El Niño events [44].

### 4.2. Sampling

High-resolution hydrographic data and water samples for phytoplankton cell determinations were collected during the research cruise “ENSO-2024” onboard the R/V “El Puma” operated by the Universidad Nacional Autónoma de México (UNAM). This cruise took place from 26 January to 10 February 2024.

The research cruise included 33 sampling stations (• symbols in Figure 1A). At each one, we recorded conductivity, temperature, and pressure throughout the water column using a CTD probe (SeaBird 9 plus) that was equipped with a fluorescence sensor (Wet Labs), and a dissolved oxygen sensor (SBE 43). All equipment was calibrated by the manufacturer prior to the cruise. Specifically, the oxygen data were further calibrated using the micro-Winkler method.

The equipment was set to acquire data at a frequency of 24 Hz, and each cast was brought as close to the bottom as possible (usually 10 m depth). Niskin bottles (10 L, General Oceanics), integrated into the CTD/Rosette system, were used to collect surface seawater samples (at 2 m depth) for the identification of phytoplankton cells and the determination of its total abundance (cells L^−1^). The water samples were immediately fixed with a Lugol–acetate solution [48] and stored in the dark until analysis in the laboratory.

### 4.3. Laboratory Analysis

After the research cruise, the samples were analyzed immediately in the laboratory. For this study, we used the Utermöhl method, employing 50 mL sedimentation columns. The sedimentation period lasted for 24 h in complete darkness [48]. Following this, the base of each column was examined under an inverted objective microscope (Carl Zeiss Axiovert A1, Oberkochen, Germany) equipped with a digital camera (Carl Zeiss Axiocam 508 color). Phytoplanktonic cells were identified down to the species level using standard identification keys [49,50], including those specific to the Pacific Ocean and Gulf of California region [51,52,53,54,55]. Finally, the counted and identified cells were standardized to total abundance units (cells L^−1^) according to the protocols outlined by Edler and Elbrachter [48].

### 4.4. Data Reduction

To determine the phase and intensity of the El Niño event, we utilized data from the Oceanic Niño Index (ONI) (https://origin.cpc.ncep.noaa.gov/ accessed on 5 March 2025). This index is widely recognized for reflecting warming or cooling events based on a threshold of ±0.5 °C anomalies in the Niño 3.4 region (5° N–5° S, 120°–170° W) [56].

The CTD data were initially converted and processed by the manufacturer’s software, averaging them at 1 dbar. Algorithms proposed by the thermodynamic equation of seawater (TEOS-10) were used to derive the conservative temperature (°C) and absolute salinity (g kg^−1^). Fluorescence data from the CTD-associated sensor were converted to chlorophyll-*a* units using the nominal file provided by the manufacturer. To analyze the vertical distribution of conservative temperature and chlorophyll-*a* within the study’s area, we selected a transect (designated as transect A-A′ in Figure 1A) that encompasses the two main regions of focus: the SGC and the ETPOM. Along this transect, the water masses were classified according to Lavín et al. [57].

Satellite data were acquired concurrently with the research cruise dates from the NASA MODIS-AQUA satellite (https://oceancolor.gsfc.nasa.gov accessed on 5 November 2024). For this study, we used level 1 and level 2 data, following the protocols outlined in Durán-Campos et al. [44]. We applied different masks/filters to eliminate false or poor-quality data. This initial processing was conducted using SeaDAS version 7.4 software, and the final maps were constructed with MATLAB R2021b routines.

Finally, to determine the influence of environmental variables on the abundance of dominant phytoplankton groups, a Canonical Correspondence Analysis (CCA) was conducted. This multivariate statistical technique enables the simultaneous examination of how different groups of organisms respond to various environmental factors [58]. We performed this analysis using the standard routines in Canoco v.4.5 software [59]. This analysis utilized two datasets: one consisting of the conservative temperature, salinity, chlorophyll-*a*, and dissolved oxygen at each sampling station and the other one containing the abundance of the dominant phytoplankton species. To reduce variance, both matrices were transformed using a square-root transformation [58].

## Data Availability

The datasets generated during this study are available from the corresponding author on request.

## Supplementary Materials

The following supporting information can be downloaded at: https://www.mdpi.com/article/10.3390/plants14091375/s1, Table S1. Species richness and total abundance (cells L^−1^) of phytoplankton in the Eastern Tropical Pacific Ocean off Mexico (ETPOM) and Southern Gulf of California (SGC) during the strong El Niño of 2023/24. Table S2. Comparison of species richness, abundance (cells L^−1^), and dominance between the Eastern Tropical Pacific Ocean off Mexico (ETPOM) and Southern Gulf of California (SGC) during the strong El Niño of 2023/2024.
