# Supplementary material for: Phytoplankton Communities in the Eastern Tropical Pacific Ocean off Mexico and the Southern Gulf of California During the Strong El Niño of 2023/24
Source: Plants (Basel). 2025 May 1;14(9):1375. doi: 10.3390/plants14091375 (PMC12073133; doi:10.3390/plants14091375)
Supplement: Supplementary file 1 [file plants-14-01375-s001.zip › Table S1.pdf]

Table S1. Species richness and total abundance (cells L^{-1}) of phytoplankton in the Eastern Tropical Pacific Ocean off Mexico (ETPOM)and Southern Gulf of California (SGC) during the strong El Niño of 2023/24.

Species	Total abundance (cells L^{-1})
**Diatoms**	
Actinoptychus adriaticus Grunow 1863	5780
Actinocyclus octonarius Ehrenberg 1837	1550
Actinocyclus senarius Ehrenberg 1838	590
Actinocyclus tenuissimus Cleve 1878	20
Alveus marinus (Grunow) Kaczmarska & Fryxell 1996	220
Amphora ovalis (Kützing) Kützing 1844	40
Asterionellopsis glacialis (Castracane) Round 1990	500
Asteromphalus arachne (Brébisson) Ralfs 1861	240
Asteromphalus cleveanus Grunow 1876	20
Asteromphalus elegans Greville 1859	60
Asteromphalus flabellatus (Brébisson) Greville 1859	20
Asteromphalus heptactis (Brébisson) Ralfs 1861	20
Asteromphalus roperianus (Greville) Ralfs 1861	40
Azpeitia nodulifera (A.W.F.Schmidt) G.A.Fryxell & P.A.Sims 1986	2660
Bacteriastrum delicatulum Cleve 1897	400
Bacteriastrum elongatum Cleve 1897	60
Bacteriastrum furcatum Shadbolt 1853	100
Bacteriastrum hyalinum Lauder 1864	580
Bacteriastrum mediterraneum Pavillard 1916	20
Bellerochea malleus (Brightwell) Van Heurck 1885	80
Biddulphia alternans (Bailey) Van Heurck 1885	20
Cerataulina dentata Hasle 1980	20
Chaetoceros aequatorialis Cleve 1901	440
Chaetoceros affinis Lauder 1864	2500
Chaetoceros atlanticus var. *skeleton* (F.Schütt) Hustedt 1930	40
Chaetoceros atlanticus var. *neapolitanus* (Schroeder) Hustedt 1930	720
Chaetoceros aurivillii Cleve 1901	80
Chaetoceros brevis F.Schütt 1895	800
Chaetoceros coarctatus Lauder 1864	20
Chaetoceros compressus Lauder 1864	40
Chaetoceros constrictus Gran 1897	40
Chaetoceros costatus Pavillard 1911	20
Chaetoceros curvisetus Cleve 1889	340
Chaetoceros danicus Cleve 1889	140
Chaetoceros decipiens Cleve 1873	2740
Chaetoceros densus (Cleve) Cleve 1899	120
Chaetoceros denticulatus H.S.Lauder 1864	340

Chaetoceros dichaeta Ehrenberg 1844	20
Chaetoceros diversus Cleve 1873	2000
Chaetoceros didymus Ehrenberg 1845	380
Chaetoceros mannaii Y.Li, Boonprakob, Moestrup & Lundholm 2018	1380
Chaetoceros messanensis Castracane 1875	800
Chaetoceros peruvianus Brightwell 1856	480
Chaetoceros protuberans Lauder 1864	140
Chaetoceros pseudocurvisetus Mangin 1910	40
Chaetoceros pseudodichaeta Ikari 1926	20
Chaetoceros radicans F.Schütt 1895	1060
Chaetoceros rectus Hernández-Becerril 1992	20
Chaetoceros salsugineus Takano 1983	40
Chaetoceros socialis H.S.Lauder 1864	1040
Chaetoceros teres Cleve 1896	840
Chaetoceros willei Gran 1897	2420
Cylindrotheca closterium (Ehrenberg) Reimann & J.C.Lewin 1964	10,950
Climacodium frauenfeldianum Grunow 1868	40
Corethron hystrix Hensen 1887	220
Corethron pennatum (Grunow) Ostenfeld 1902	140
Coscinodiscus asteromphalus Ehrenberg 1844	360
Coscinodiscus centralis Ehrenberg 1839	100
Coscinodiscus granii L.F.Gough 1905	1140
Coscinodiscus radiatus Ehrenberg 1840	20
Coscinodiscus wailesii Gran & Angst 1931	360
Cyclotella stylorum Brightwell 1860	680
Cyclotella choctawhatcheeana Prasad 1990	300
Cyclotella litoralis Lange & Syvertsen 1989	3060
Dactyliosolen fragilissimus (Bergon) Hasle 1996	160
Dactyliosolen mediterraneus (H.Peragallo) H.Peragallo 1892	1220
Dactyliosolen phuketensis (B.G.Sundström) G.R.Hasle 1996	680
Detonula moseleyana (Castracane) H.H.Gran 1900	1140
Detonula pumila (Castracane) Gran 1900	640
Ditylum brightwellii (T.West) Grunow 1885	200
Eucampia cornuta (Cleve) Grunow 1883	9580
Eucampia zodiacus Ehrenberg 1839	180
Eupyxidicula palmeriana (Greville) S.Blanco & C.E. Wetzel 2016	20
Eupyxidicula turris (Greville) S.Blanco & C.E.Wetzel 2016	180
Fragilariopsis doliolus (Wallich) Medlin & P.A.Sims 1993	1720
Fragilariopsis pseudonana (Hasle) Hasle 1993	20
Grammatophora marina (Lyngbye) Kützing 1844	40
Guinardia cylindrus (Cleve) Hasle 1996	40
Guinardia flaccida (Castracane) H.Peragallo 1892	840
Guinardia striata (Stolterfoth) Hasle 1996	5020
Gyrosigma spenceri (Bailey ex Quekett) Griffith & Henfrey 1856	40
Hobaniella longicruris (Greville) P.A.Sims & D.M.Williams 2018	180

Haslea trompii (Cleve) Simonsen 1974	720
Hemiaulus hauckii Grunow ex Van Heurck 1882	660
Hemiaulus membranaceus Cleve 1873	480
Hemiaulus sinensis Greville 1865	20
Lauderia annulata Cleve 1873	100
Leptocylindrus danicus Cleve 1889	1320
Leptocylindrus minimus Gran 1915	140
Licmophora flabellata (Greville) C.Agardh 1831	7460
Lioloma pacificum (Cupp) Hasle 1996	2000
Mastogloia cribosa Grunow 1860	20
Melosira varians C.Agardh 1827	20
Meuniera membranacea (Cleve) P.C.Silva 1996	600
Navicula directa (W.Smith) Brébisson 1854	20
Navicula distans (W.Smith) Brébisson 1854	20
Neodelphineis pelagica H.Takano, nom. inval. 1983	560
Nitzschia bicapitata Cleve 1901	1260
Nitzschia ikeanae Fryxell & H.Y.Lee 1996	100
Nitzschia interruptestriata Simonsen 1974	160
Nitzschia leehyi G.Fryxell 2000	20
Nitzschia longissima (Brébisson ex Kützing) Grunow 1862	1920
Nitzschia sicula (Castracane) Hustedt 1958	40
Pachyneis gerlachii Simonsen 1974	120
Planktoniella muriformis (Loeblich III, W.W.Wight & W.M.Darley) Round 1972	1840
Planktoniella sol (G.C.Wallich) Schütt 1892	2440
Pleurosigma decorum W.Smith 1853	320
Pleurosigma nicobaricum Grunow 1880	40
Pleurosigma normanii Ralfs 1861	260
Pleurosigma salinarum (Grunow) Grunow 1880	20
Proboscia alata (Brightwell) Sundström 1986	1120
Proboscia indica (H.Peragallo) Hernández-Becerril 1995	20
Pseudoguinardia recta von Stosch 1986	180
Pseudo-nitzschia americana (Hasle) Fryxell 1993	60
Pseudo-nitzschia brasiliana Lundholm, Hasle & G.A.Fryxell 2002	120
Pseudo-nitzschia multistriata (H.Takano) H.Takano 1995	20,710
Pseudo-nitzschia pseudodelicatissima (Hasle) Hasle 1993	57,000
Pseudo-nitzschia pungens (Grunow ex Cleve) Hasle 1993	35,460
Pseudo-nitzschia roundii D.U.Hernández-Becerril 2006	3500
Pseudo-nitzschia sabit S.T.Teng, H.C.Lim, P.T.Lim & C.P.Leaw 2015	100
Pseudo-nitzschia subfraudulenta (Hasle) Hasle 1993	680
Pseudo-nitzschia subpacifica (Hasle) Hasle 1993	260
Ralfsiella smithii (Ralfs) P.A.Sims, D.M.Williams & Ashworth 2018	20
Rhizosolenia acuminata (H.Peragallo) H.Peragallo 1907	80
Rhizosolenia bergonii H.Peragallo 1892	480
Rhizosolenia clevei Ostenfeld 1902	40
Rhizosolenia fallax B.G.Sundström 1986	60

Rhizosolenia imbricata Brightwell 1858	20
Rhizosolenia ostenfeldii B.G.Sundström 1986	20
Rhizosolenia pungens A.Cleve 1937	400
Rhizosolenia temperei H.Peragallo 1888	20
Roperia tesselata (Roper) Grunow ex Pelletan 1889	230
Shionodiscus oestrupii (Ostenfeld) A.J.Alverson, S.-H.Kang & E.C.Theriot 2006	20
Skeletonema pseudocostatum Medlin 1991	640
Skeletonema tropicum Cleve 1900	1920
Stigmaphora lanceolata Wallich 1860	70
Stigmaphora rostrata Wallich 1860	40
Surirella fastuosa (Ehrenberg) Ehrenberg 1843	160
Tetramphora decussata (Grunow) Stepanek & Kociolek 2016	60
Thalassionema frauenfeldii (Grunow) Tempère & Peragallo 1910	880
Thalassionema nitzschioides (Grunow) Mereschkowsky 1902	1160
Thalassiosira angustelineata (A.W.F.Schmidt) G.Fryxell & Hasle 1977	100
Thalassiosira diporocyclus Hasle 1972	20
Thalassiosira eccentrica (Ehrenberg) Cleve 1904	840
Thalassiosira echinata H.J.Semina 1994	20
Thalassiosira leptopus (Grunow) Hasle & G.Fryxell 1977	100
Thalassiosira punctifera (Grunow) Fryxell, Simonsen & Hasle 1974	20
Thalassiosira tealata H.Takano 1980	1180
Thalassiosira tenera Proshkina-Lavrenko 1961	3240
Trieres mobiliensis (Bailey) Ashworth & E.C.Theriot 2013	20
**Dinoflagellates**	
Achradina pulchra Lohmann 1903	100
Akashiwo sanguinea (K.Hirasaka) Gert Hansen & Moestrup 2000	40
Alexandrium minutum Halim 1960	100
Alexandrium tamiyavanichii Balech 1994	20
Amphisolenia bidentata B.Schröder 1900	20
Azadinium caudatum (Halldal) Nézan & Chomérat 2012	180
Azadinium poporum Tillmann & Elbrächter 2011	540
Azadinium spinosum Elbrächter & Tillmann 2009	160
Blepharocysta denticulata D.-S.Nie 1939	3320
Blepharocysta paulsenii Schiller 1937	240
Blepharocysta splendor-maris (Ehrenberg) Ehrenberg 1873	240
Boreadinium breve (T.H.Abé) Sournia 1984	20
Ceratoperidinium falcatum (Kofoid & Swezy) Reñé & Salas 2013	20
Corythodinium biconicum (Kofoid) F.J.R.Taylor 1976	40
Corythodinium reticulatum (Stein) F.J.R.Taylor 1976	40
Corythodinium tesselatum (F.Stein) Loeblich Jr. & Loeblich III 1966	120
Cucumeridinium coeruleum (Dogiel) F.Gomez, P.López-García, H,Takayama & D.Moreira 2015	4400
Cucumeridinium lira (Kofoid & Swezy) F.Gómez, P. López-García, H.Takayama & D.Moreira 2015	80
Dinophysis caudata Kent 1881	60

Diplopelta asymmetrica (Mangin) M.Lebour ex Balech 1988	400
Diplopelta globula (T.H.Abé) Balech 1979	80
Diplopsalopsis orbicularis (Paulsen) Meunier 1910	40
Fragilidium mexicanum Balech 1988	120
Gonyaulax areolata Kofoid & J.R.Michener 1911	40
Gonyaulax diegensis Kofoid 1911	20
Gonyaulax fragilis (Schütt) Kofoid 1911	20
Gonyaulax fusiformis H.W.Graham 1942	40
Gonyaulax polygramma F.Stein 1883	20
Grammatodinium tongyeonginum Z.Li & H.H.Shin 2017	40
Gymnodinium catenatum H.W.Graham 1943	1140
Gymnodinium impudicum (S.Fraga & I.Bravo) Gert Hansen & Moestrup 2000	40
Gyrodinium fusiforme Kofoid & Swezy 1921	16,100
Gyrodinium pepo (F.Schütt) Kofoid & Swezy 1921	20
Gyrodinium rubrum (Kofoid & Swezy) Y.Takano & T.Horiguichi 2004	120
Gyrodinium spirale (Bergh) Kofoid & Swezy 1921	1120
Heterocapsa niei (A.R.Loeblich) L.C.Morrill & A.R.Loeblich 1981	280
Heterocapsa orientalis Iwataki, Botes & Fukuyo 2003	3640
Kapelodinium vestifici (Schütt) Boutrup, Moestrup & Daugbjerg 2016	10,840
Karenia bicuneiformis Botes, Sym & Pitcher 2003	960
Karenia brevisulcata (F.H.Chang) Gert Hansen & Moestrup 2000	6640
Karenia mikimotoi (Miyake & Kominami ex Oda) Gert Hansen & Moestrup 2000	100
Karenia selliformis A.J.Haywood, K.A.Steidinger & L.MacKenzie 2004	560
Levanderina fissa (Levander) Moestrup, Hakanen, Gert Hansen, Daugbjerg & M.Ellegaard 2014	20
Lingulodinium polyedra (F.Stein) J.D.Dodge 1989	760
Margalefidinium fulvescens (M.Iwataki, H.Kawami & Matsuoka) F.Gómez, Richlen & D.M.Anderson 2017	20
Margalefidinium polykrikoides (Margalef) F.Gómez, Richlen & D.M.Anderson 2017	20
Nematodinium armatum (Dogiel) Kofoid & Swezy 1921	680
Noctiluca scintillans (Macartney) Kofoid & Swezy 1921	20
Ornithocercus magnificus F.Stein 1883	20
Oxyphysis oxytoxoides Kofoid 1926	20
Oxytoxum curvatum (Kofoid) Kofoid & J.R.Michener 1911	60
Oxytoxum mediterraneum Schiller 1937	1060
Oxytoxum sceptrum (F.Stein) Schröder 1900	1960
Oxytoxum scolopax F.Stein 1883	360
Oxytoxum variabile J.Schiller 1937	1440
Phalacroma cuneus F.Schütt 1895	20
Phalacroma parvulum (Schütt) Jørgensen 1923	40
Phalacroma rotundatum (Claparéde & Lachmann) Kofoid & J.R.Michener 1911	60
Podolampas bipes F.Stein 1883	40
Podolampas palmipes F.Stein 1883	60
Podolampas spinifera Okamura 1912	100
Preperidinium meunieri (Pavillard) Elbrächter 1993	240

Pronoctiluca spinifera (Lohmann) Schiller 1932	1040
Prorocentrum compressum (Bailey) T.H.Abé ex J.D.Dodge 1975	4920
Prorocentrum gracile F.Schütt 1895	1380
Prorocentrum koreanum M.-S.Han, S.Y.Cho & P.Wang 2016	100
Prorocentrum lenticulatum (Matzenauer) F.J.R.Taylor 1976	1500
Prorocentrum mexicanum Osorio-Tafall 1942	280
Prorocentrum obtusidens J.Schiller 1928	3620
Prorocentrum robustum Osorio-Tafall 1942	360
Prorocentrum rostratum F.Stein 1883	40
Prorocentrum sigmoides Böhm 1933	80
Protoperidinium bispinum (J.Schiller) Balech 1974	300
Protoperidinium brochii (Kofoid & Swezy) Balech 1974	20
Protoperidinium cassum (Balech) Balech 1974	100
Protoperidinium cepa (Balech) Balech 1974	20
Protoperidinium claudicans (Paulsen) Balech 1974	40
Protoperidinium corniculum (Kofoid & J.R.Michener) F.J.R.Taylor & Balech 1988	20
Protoperidinium depressum (Bailey) Balech 1974	20
Protoperidinium furcatum (T.H.Abé) Balech 1994	60
Protoperidinium marukawae (T.H.Abé) Balech 1974	140
Protoperidinium mastophorum (Balech) Balech 1974	40
Protoperidinium mite (Pavillard) Balech 1974	20
Protoperidinium obtusum (Karsten) Parke & J.D.Dodge 1976	220
Protoperidinium oceanicum (Vanhöffen) Balech 1974	20
Protoperidinium ovum (J.Schiller) Balech 1974	120
Protoperidinium punctulatum (Paulsen) Balech 1974	11,320
Protoperidinium robustum (Meunier) Hernández-Becerril 1991	6300
Protoperidinium steinii (Jørgensen) Balech 1974	140
Protoperidinium tuba (J.Schiller) Balech 1974	320
Pyrocystis fusiformis C.W.Thomson 1876	60
Pyrocystis lunula (F.Schütt) F.Schütt 1896	40
Pyrocystis pseudonoctiluca Wyville-Thompson 1876	40
Pyrophacus steinii (Schiller) Wall & Dale 1971	940
Scrippsiella acuminata (Ehrenberg) Kretschmann, Elbrächter, Zinssmeister, S.Soehner, Kirsch, Kusber & Gottschling 2015	3820
Scrippsiella spinifera G.Honsell & M.Cabrini 1991	260
Spiraulax kofoidii H.W.Graham, nom. illeg. 1942	20
Thoracosphaera heimii (Lohmann) Kamptner 1944	940
Torquentidium convolutum (Kofoid & Swezy) H.H.Shin, Z,Li, K.W.Lee & K.Matsuoka 2019	40
Torquentidium helix (Lemmermann) H.H.Shin, Z.Li, K.W.Lee & K.Matsuoka 2019	620
Triadinium polyedricum (Pouchet) J.D.Dodge 1981	5320
Tripos brevis (Ostenfeld & Johannes Schmidt) F.Gómez 2021	20
Tripos falcatus (Kofoid) F.Gómez, nom. inval. 2013	20
Tripos furca (Ehrenberg) F.Gómez 2013	280
Tripos fusus (Ehrenberg) F.Gómez 2013	80

Tripos humilis (Jørgensen) F.Gómez 2013	60
Tripos kofoidii (Jørgensen) F.Gómez 2013	80
Tripos lineatus (Ehrenberg) F.Gómez 2021	100
Tripos macroceros (Ehrenberg) Hallegraeff & Huisman 2020	60
Tripos muelleri Bory 1826	20
Tripos teres (Kofoid) F.Gómez 2013	20
Tripos trichoceros (Ehrenberg) Gómez 2013	20
**Silicoflagellates**	
Octactis octonaria (Ehrenberg) Hovasse 1946	760
Dictyocha calida Poelchau 1976	180
Dictyocha californica Schrader & Murray 1985	80
Dictyocha fibula Ehrenberg 1839	460
Dictyocha fibula var. *robusta* Schrader & Murray	20
**Ciliates**	
Mesodinium rubrum Lohmann 1908	20,980
**Cynobacteria**	
Trichodesmium hildebrandtii Gomont 1892	1760
