# Supplementary material for: Phytoplankton Communities in the Eastern Tropical Pacific Ocean off Mexico and the Southern Gulf of California During the Strong El Niño of 2023/24
Source: Plants (Basel). 2025 May 1;14(9):1375. doi: 10.3390/plants14091375 (PMC12073133; doi:10.3390/plants14091375)
Supplement: Supplementary file 1 [file plants-14-01375-s001.zip › Table S2.pdf]

**Table S2.** Comparison of species richness, abundance (cells $L^{-1}$), and dominance between the Eastern Tropical Pacific Ocean off Mexico (ETPOM) and Southern Gulf of California (SGC) during the strong El Niño of 2023/2024.

| | **Eastern Tropical Pacific Ocean off Mexico** | | | **Southern Gulf of California** | | |
|---|---|---|---|---|---|---|
| | **Species richness** | **Abundance (cells $L^{-1}$)** | **Dominant specie (abundance)** | **Species richness** | **Abundance (cells $L^{-1}$)** | **Dominant specie** |
| **Diatoms** | 86 | 20,200 | *Guinardia striata* (Stolterfoth) Hasle 1996 (3060 cells $L^{-1}$) | 137 | 204,700 | *Pseudo-nitzschia pseudodelicatissima* (Hasle) Hasle 1993 (55,300 cells $L^{-1}$) |
| **Dinoflagellates** | 69 | 37,000 | *Gyrodinium fusiforme* Kofoid & Swezy 1921 (5460 cells $L^{-1}$) | 99 | 67,520 | *Gyrodinium fusiforme* Kofoid & Swezy 1921 (10,640 cells $L^{-1}$) |
| **Silicoflagellates** | 4 | 260 | *Dictyocha fibula* Ehrenberg 1839 (160 cells $L^{-1}$) | 4 | 1240 | *Octactis octonaria* (Ehrenberg) Hovasse 1946 (700 cells $L^{-1}$) |
| **Ciliates** | 1 | 6860 | *Mesodinium rubrum* Lohmann 1908 (6860 cells $L^{-1}$) | 1 | 14,120 | *Mesodinium rubrum* Lohmann 1908 (14,120 cells $L^{-1}$) |
| **Cyanobacteria** | 1 | 980 | *Trichodesmium hildebrandtii* Gomont 1892 (980 cells $L^{-1}$) | 1 | 780 | *Trichodesmium hildebrandtii* Gomont 1892 (780 cells $L^{-1}$) |
